# Comparative efficacy of esomeprazole and omeprazole: Racemate to single enantiomer switch

**DOI:** 10.1186/s40199-015-0133-6

**Published:** 2015-11-14

**Authors:** Waheed Asghar, Elliot Pittman, Fakhreddin Jamali

**Affiliations:** Faculty of Pharmacy and Pharmaceutical Sciences, University of Alberta, 11361 – 87 Avenue, Edmonton, AB T6G 2E1 Canada

**Keywords:** Omeprazole, Esomeprazole, Enantiomer, GERD, Acid control, *H. pylori*, Comparative efficacy

## Abstract

**Background:**

Both omeprazole and its S enantiomer (esomeprazole) have been available and used to treat  symptoms of gastroesophageal reflux disease (GERD) and conditions associated with excessive stomach acid secretion for more than a decade. Controversy exists over improved efficacy of S enantiomer (esomeprazole) over parent racemate (omeprazole). However, a comparison of the clinical outcomes of these products may reveal the rationale for switching from the racemate to single enantiomer. Since enantiomers of omeprazole are equipotent, we compared the outcomes of equal doses of each product to see if both actually differ in their efficacy’s or the reported superiority of S enantiomer is just a dose effect.

**Methods:**

A web search was carried out for randomized controlled trials with head-to-head comparisons of omeprazole and S-omeprazole. The data were abstracted and after calculating theodd ratios (OR) for the outcomes reported in each study, the combined overall odd ratios (OR’) were estimated. The random effect inverse variance method with omeprazole as the reference (OR” = 1) was used.

**Results:**

Out of 1171 studies, 14 were deemed eligible. There was no significant difference in the therapeutic success between omeprazole and S-omeprazole as a part of triple therapy for the treatment of H. pylori in both intention-to-treat (OR’, 1.06; CI, 0.83, 1.36; *p =* 0.63) as well as per-protocol analysis (OR’, 1.07; CI, 0.84, 1.36; *p =* 0.57). For the treatment of gastro-oesophageal reflux disease, S-omeprazole was significantly but marginally superior to the racemate (OR’, 1.18; CI, 1.01, 1.38; *p =* 0.04). The two products were equipotent in all metrics used to assess intragastric pH except for the % patients maintaining a 24 h gastric pH above 4 (1.57; CI, 1.04, 2.381; *p =* 0.03).

**Conclusion:**

The therapeutic benefit of chiral switch of omeprazole is questionable considering the substantially greater economic burden involved.

## Background

Stereochemical aspects of drug actions and drug disposition have become a subject of interest since the early 1980s [1]. Most chiral drugs have been used as racemates while the beneficial effects are often attributed mainly to one of the enantiomers. Hence, it was intuitively believed that a product containing the stereochemically pure enantiomer with the main pharmacological activity would be superior to its racemate counterpart. This overwhelming notion has not been without opposition due to increased toxicity risk in humans [2] or in experimental animal [3–5]. Nevertheless, many attempts have been made during the past decades to switch from racemate to stereochemically pure drug products. This has resulted in the introduction of a few products in the past decade, e.g., levofloxacin, dexibuprofen and esomeprazole. At the time of their development, reasonable rationales for such a switch had been offered without unequivocal data on the superiority of the single enantiomers. For example, omeprazole and S-omeprazole have been the subject of many randomized controlled trials (RCTs) and , cohort and case-control studies. However, these studies and the subsequent systematic reviews [6–12], typically compared non-comparable doses, i.e., 40 mg S-omeprazole vs 20 mg racemic omeprazole. In addition, recently, Gellad et al., who reviewed 4 RCTs that included non-comparative doses, concluded mixed evidence for the superiority of the single S enantiomer over the racemate [13]. Thus, a comparison of the available data on comparative doses is needed. From the pharmacological viewpoints, the drug is not stereoselective since its properties are attributed to both enantiomers [14]. Its pharmacokinetics, on the other hand, are stereoselective with the S enantiomer having a higher bioavailability yielding a greater body exposure than R-omeprazole. A 40 mg dose of S-omeprazole, therefore, yields greater than twice the body exposure than a 20 mg dose of the racemate. Thus, clinical trials that have compared 40 vs 20 mg have not assessed comparative doses. Only, a 2005 meta-analysis that focused on *Helicobacter pylori* (*H. pylori*) eradication had included a comparison of equal doses of the racemate and S-omeprazole [15]. The purpose of this work was to analyse all clinical data on the efficacy of equal mg doses of S-omeprazole versus that of racemic omeprazole reported until April 2015. This is with the realization that a dose of the single S enantiomer will result in a greater body exposure when compared to an equal mg dose of the racemate. We stratified the data based on therapeutic, symptomatic and intragastric pH control outcomes. We also analyzed available data based on the type of analysis used; i.e., intention-to-treat and per-protocol. In addition, we assessed the dose-dependency of omeprazole effects.

## Methods

### Literature search

A web search was conducted using a set of keywords (Appendix 1), in databases including MEDLINE (Medical Literature Analysis and Retrieval System Online database), EMBASE (Excerpta Medica database), CINHAL (Cumulative Index to Nursing and Allied Health Literature), IPA (International Pharmaceutical Abstracts), PASCAL (Dedicated Database for European Science, Technology and Medicine), Cochrane, EBM (The Evidence-Based Medicine database) and Google Scholar. We looked for studies reporting comparative RCTs published until April 2015. The United States Food and Drug Administration (FDA) and pharmaceutical manufacturer’s websites were also searched for any relevant literature. Reference lists from review articles were also checked for any relevant information, if available. Two reviewers (W.A. and E.P) independently reviewed the studies for the inclusion and exclusion criteria and conflicts were resolved by mutual agreement.

### Data analysis

The data from eligible studies was abstracted and analysed according to published methods [16] and the odds ratios (ORs) of each study were manually calculated for each outcome including: (i) therapeutic success; i.e., as part of triple therapy for eradication of H. pylori or healing of esophagitis or peptic ulcer; (ii) symptomatic relief of heart burn, (gastro-oesophageal reflux disease, GERD; (iii) % of patient with median 24 h intragastric pH above 4. The calculated OR values from all studies were then merged to create the combined odds ratio (OR’) using the Review Manager software recommended by Cochrane, and employing the random effect inverse variance method [17]. For cross-over studies, odd ratios were calculated based on the matched samples case–control approach [18]. We chose omeprazole to be the reference (OR’ = 1). For metrics that OR could not be calculated (i.e., median pH in 24 h, mean time pH > 4 and % of 24 h with pH > 4) actual measured values were used to assess the differences.

### Selection criteria

Only RCTs carried out in an adult population (>18 years age) having both S-omeprazole and omeprazole, in head to head comparisons, at equivalent oral doses, and published in English were included in our analysis. No outcome restriction was considered at this stage. All formulations (capsule, tablet, and suspension, both immediate and delayed release) with approved doses, regimens, with any salt (magnesium/strontium/sodium), and for any duration of treatment were considered eligible. Both the intention-to-treat (all data included regardless of whether or not they completed or received that treatment) and per-protocol studies were included and analysed separately.

Any study conducted in a paediatric population (age < 18 years), comparing inequivalent doses (40 mg vs. 20 mg), or administering the drug by any route other than per-oral were excluded from our analysis. Additionally, studies reporting the use of more than two acid suppressing agents and/or had drug/brand switching during the trial were also excluded.

### Heterogeneity

The variability in outcomes measure (i.e., heterogeneity of analysis) was determined using Cochrane’s Q and the I2 statistics [19] as reported here (Table 1). The methodological quality of all eligible studies was assessed using the previously published Newcastle-Ottawa scale with scores >5 deemed as acceptable. All eligible studies scored between 6 and 7 [20].

**Table 1:**
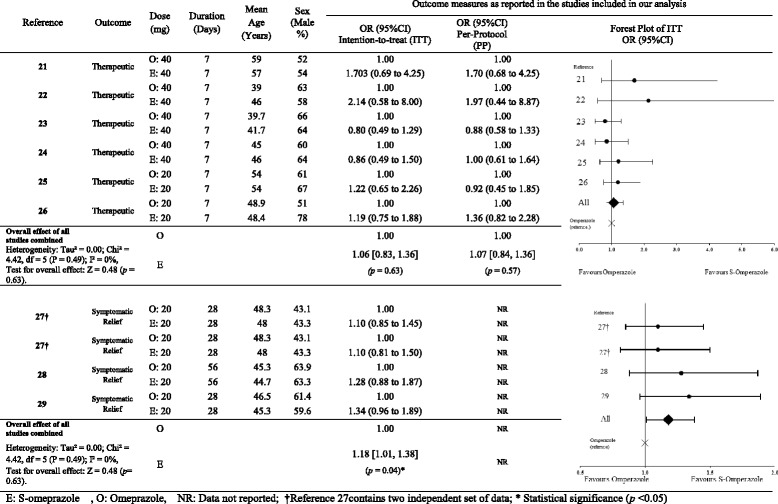
Characteristics of the studies and odds ratio OR (95 % CI) for studies reporting therapeutic and symptomatic relief outcomes

### Strengths and weaknesses

To the best of our knowledge, this systematic review presents the only exhaustive and up-to-date analysis of the efficacy of omeprazole (racemate) and esomeprazole (S enantiomer) at equivalent doses. We have also provided a comprehensive analysis of all outcomes reported in the included trials. The fact that most of the eligible studies were sponsored by the maker of the drugs is the limitation of our study. Availability and analysis of data on the prophylactic potential of these drugs would have been further useful and informative.

## Results

**Fig. 1:**
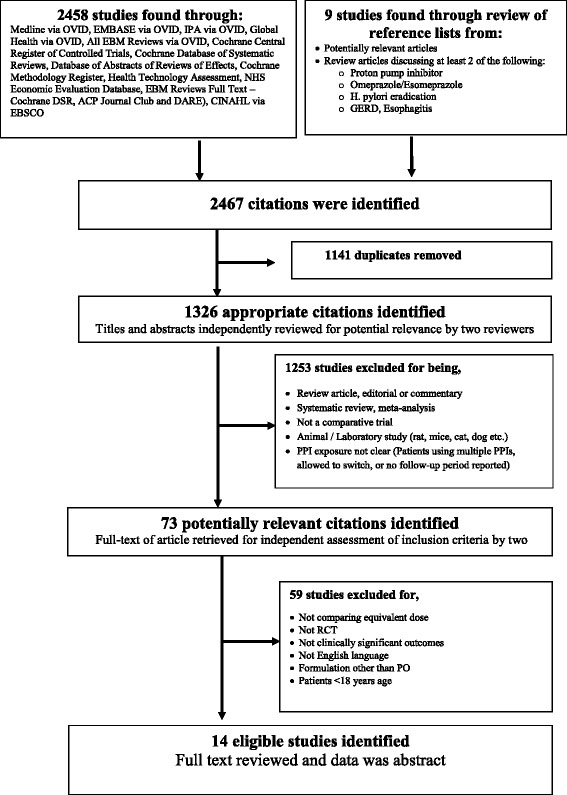
Flow diagram of the selection process for randomized controlled trials reporting omeprazole vs esomeprazole (Published until April 2015)

Our search yielded 2467 studies of which, after review of the title and abstract, 73 were deemed potentially relevant. These studies were retrieved in full text and reviewed (Fig. 1). Of those, only 14 studies were deemed eligible after full review [21–34] (Tables 1, 2, 3).

**Table 2:** Characteristics of the studies and odds ratio OR (95%CI) for studies reporting 24 h median intra-gastric pH as outcomes

Reference	Dose (mg)	Duration (Days)	Mean Age (Years)	Sex (Male %)	Outcome measures as reported in the studies included in our analysis			
					Odds of 24 h median intra-gastric pH > 4 OR	Median intra-gastric pH within 24 h post dose (pH)	Mean time pH > 4 within 24 h post dose (h)	% time duration of 24 h with intra-gastric pH > 4 (%)
30	O:40	1	31.7	46	1.00	4.5 (4.36- 4.64)	17.8 (17.4-18.5)	62.0 (59.0-65.0)
	E: 40	1	31.7	46	2.08 (1.10, 3.96)	4.8 (4.64- 4.92)	19.2 (18.6-19.75)	68.4 (65.4-71.4)
31	O:20	1	58	47	NR	6.4 (6.32- 6.42)	NR	NR
	E: 20	1	59	47	NR	6.4 (6.30- 6.52)	NR	NR
32	O:20	5	45	42	1.00	3.6 (3.2- 3.9)	10.5 (8.8-12.2)	43.7 (36.7-50.7)
	E: 20	5	45	42	1.23 (0.63, 2.38)	4.1 (3.8- 4.5)	12.7 (11.0-14.4)	53.0 (46.0-60.0)
33	O:20	7	21.7	75	NR	5.4 (3.5 - 6.8)	22.6 (20.3–24)]	79.2 (40.0-90.2)
	E: 20	7	21.7	75	NR	5.4 (3.5–6.8)	21.1 (17.2–23.8)	81.0 (60.0-90.0)
34	O:20	5	18-6	46	1.00	3.5 (1.6-5.3)	10.4 (3.0–20.2)	44.0 (12.4-83.9)
	E:20	5	18-6	46	1.42 (0.56, 3.63)	3.9 (1.9-5.1)	11.3 (3.7–18.0)	48.0 (15.5-75.3)
Overall effect of all studies combined:	O				1.00	4.39 (3.36, 5.73)	15.24 (12.13, 19.14)	52.01 (39.52, 68.44)
	E				1.57 (1.04, 2.38) (*p* = 0.03)*	4.69 (3.79, 5.81) (*p* = 0.67)	16.43 (13.72, 19.66) (*p* = 0.55)	60.10 (48.58, 74.34) (*p* = 0.40)

* Statistical significance of difference from referance (*p*  < 0.05), NR- Data not reported

**Table 3:** The effect of 20 mg and 40 mg doses of omeprazole and S-omeprazole

Drug and Dosage	Therapeutic outcome	Intra-gastric pH outcome			
	% of patients cured (i.e. treatment of H. pylori) Mean % (SD)	% of patients cured (i.e. 24 h intra-gastric pH >4) Mean % (SD)	Median intra-gastric pH within 24 h post dose Mean pH (SD)	Mean time pH > 4 within 24 h post dose Mean h (SD)	% time duration of 24 h with intra-gastric pH > 4 Mean % (SD)
Omeprazole (O 20)	80.0 (11.3), *n* = 2	37.5 (9.2), *n* = 2	4.7 (1.4), *n* = 4	14.5 (7.0), *n* = 3	55.6 (20.4), *n* = 3
Omeprazole (O 40)	79.8 (7.1), *n* = 4	75.0^a^ *n* = 1	4.5^a^ *n* = 1	17.8^a^ *n* = 1	62.0^a^ *n* = 1
S-Omeprazole (E 20)	83.0 (11.3) *n* = 2	49.0 (7.1), *n* = 2	4.9 (1.2), *n* = 4	15.0 (5.3), *n* = 3	60.7 (17.8), *n* = 3
S-Omeprazole (E 40)	84.0 (7.6) *n* = 4	88.0^a^ *n* = 1	4.8^a^ *n* = 1	19.2^a^ *n* = 1	68.4^a^ *n* = 1

^a^No variance since *n* = 1; NR- Data not reported

Our final selection included 6 studies [21–26] (Table 1) reporting the treatment of peptic ulcer secondary to *H. pylori* infection with omeprazole or S-omeprazole as part of a 7-day triple therapy. Four of these studies [21–24] compared 40 mg daily doses while the other two [25, 26] used 20 mg daily doses of the two products. Three studies [27–29] (Table 1) were analysed that included data on the relief from GERD offered by omeprazole versus S-omeprazole. Five studies were included which reported [30–34] 24 h median intragastric pH values after administration of omeprazole and S-omeprazole.

There was no significant difference in the therapeutic success between omeprazole and S-omeprazole as a part of triple therapy for the treatment of *H. pylori* in both intention-to-treat (OR’, 1.06; CI, 0.83, 1.36; *p =* 0.63; *n* = 6) as well as per-protocol analysis (OR’, 1.07; CI, 0.84, 1.36; *p =* 0.57; *n* = 6). Data for per-protocol analysis were only available for *H. pylori* treatment.

For the treatment of GERD, however, S-omeprazole was found to be marginally superior to omeprazole (OR’, 1.18; CI, 1.01, 1.38; *p =* 0.04; *n* = 3).

Among the metrics used to compare the effectiveness of the two products to control intragastric pH (Table 2), only the percent patients maintaining a 24 h gastric pH above 4 was significantly greater for S-omeprazole as compared with racemic omeprazole (OR’: 1.57; CI, 1.04, 2.381; *p =* 0.03; *n* = 3). For other pH metrics [35], we found 5 studies that included outcomes of the median intragastric pH, duration of intragastric pH > 4, and percent of patients having intragastric pH > 4 during the 24 h post dose [30–34].

## Discussion

Omeprazole is a racemic drug with both enantiomers entering the parietal cells where, in the presence of acid, they are converted to an achiral sulphenamide that, in turn, inhibits the proton pumps therein [36]. The pharmacological effects of omeprazole are, therefore, not stereoselective [14]. Its pharmacokinetics, on the other hand, are stereoselective. Upon its rapid absorption, the drug undergoes a stereoselective first-pass metabolism mediated by CYP2C19 in favour of the R enantiomer. For switching from racemic omeprazole to its S enantiomer, the following rationale were offered [37] (i) omeprazole controls intragastric pH for only 10 h while the S-enantiomer does so for a longer period; (ii) an increase in dose, does not add to the beneficial effects of the racemate but it does so with the S-enantiomer; (iii) there is a less inter-subject variability in response to S-omeprazole as compared to the racemate.

Our analysis reveals that, indeed, there is no significant difference between the two products in the duration of pH control (Table 2). Indeed, the two products were equally effective in terms of other pH related outcomes except for the effectiveness to maintain the value above 4 for which the OR’ was greater for S-omeprazole as compared with the racemate.

Some investigators have compared the therapeutic outcomes of the recommended doses of the two drugs; i.e., 20 mg omeprazole vs 40 mg S-omeprazole. Thus, since the enantiomers of omeprazole are equipotent, the comparison has been made between 20 and 40 mg of the active compound. In addition, since the R enantiomer undergoes a greater extent of first-pass metabolism, and the S enantiomer has a nonlinear pharmacokinetics, the body exposure of 40 mg S-omeprazole is expected to be even greater than twice that of 20 mg racemic omeprazole. These studies [6–12], with one exception [15], have reported a greater beneficial effect for 40 mg doses of S-omeprazole as compared to 20 mg of the racemic drug. However, our analysed of the available data revealed no differences between 20 and 40 mg of either omeprazole or S-omeprazole with respect to both therapeutic and pH control outcomes (Table 3). This is despite the fact that a 40 mg dose of S-omeprazole is expected to yield a substantially greater drug bioavailability than a 20 mg racemate or single enantiomer [37]. This suggests that the examined dosage range may be at the plateau phase of the dose-effect curve. We were unable to find data comparing the effect of dose elevation on GERD.

Our analysis revealed a marginal but significantly greater effect in the control of GERD for S-omeprazole (OR’, 1.18; CI, 1.01, 1.38) as compared to omeprazole (reference, OR 1.0) (Table 1). This difference, although statistically significant, may be of questionable therapeutic value as the OR’ is calculated to be very close to unity.

The link between plasma omeprazole concentration and its beneficial effects is complicated and mainly unknown. The drug has an apparent plasma t1/2 of approximately 1 h but a duration of effect of 72 h [38]. Drawing therapeutic inferences based merely on the pharmacokinetics properties alone and in the absence of a clear understanding of the kinetics of pharmacological actions is questionable. It is clear that at the time of drug development, some advantages were speculated, however, due to the emergence of more information over the past decade, a more reliable analysis of the data has become possible. We can now conclude more conclusively that despite the overwhelming economic success of S-omeprazole, the drug offers little or no advantage over its parent racemic product.

Despite the lack of success in therapeutic outcome, the S enantiomer of omeprazole has been mentioned, particularly in public and trade media, as an example of racemic to enantiomer switch success. The market success of the switch cannot be disputed due to the ever-growing market share of the acid-controlling agent (approximately $5 billion in 2013) [39]. This is significant as the monthly cost of S-omeprazole is up to over ten-fold of that of omeprazole.

The advances in stereochemical aspects of drug action and disposition have enhanced our understanding of the mechanisms behind both the beneficial and harmful outcomes of drugs. For example, we have reported that inflammatory disease slows down clearance of racemic verapamil. The extent of this drug-disease interaction is only 3-fold based on achiral analysis but 11-fold when the S enantiomer is considered [40]; pharmacological properties of verapamil are mainly attributed to its S enantiomer. In addition, the well-known enantiomeric bioconversion of some drugs has significantly added to the knowledge of the field [41, 42] so that many pharmaceutical houses were prompted to develop new drugs as stereochemically pure products, or to consider the racemic-enantiomer switch of available drugs, though there have been very few successful results [13]. Based on some data generated using animal models, we have reported that the stereochemically pure enantiomers of the racemic nonsteroidal anti-inflammatory drugs do not provide safer alternatives with regard to the well-known gastrointestinal side effects of these drugs [3–5]. In addition, for ofloxacin to levofloxacin and ibuprofen to dexibuprofen switching, (i.e., another two successful racemic to enantiomer switches) there are no comparative data available to assess the claimed superiority of one over the other. Altogether, it is reasonable to suggest that, despite the earlier intuitive belief, stereochemically pure drugs are not necessarily superior to their corresponding racemates [3]. This by no means implies that the stereochemical aspects of a drug’s action and dispositions are not of prime importance in clinical pharmacology or toxicology research.

## Conclusion

Overall S-omeprazole appeared to be as effective as omeprazole when used at equivalent doses in treating ulcers as part of triple therapy, and in controlling 24 h intragastric pH. For both omeprazole and S-omeprazole the differences between 20 and 40 mg doses, if any, are marginal.

## Appendix 1: List of search terms and key words used

1. exp omeprazole sulfone/or exp omeprazole/or exp omeprazole derivative/or omeprazole.mp.
2. omepr$.mp.
3. (Antra or Aspra or Gastroloc or Losectil or Lozeprel or Mopral or Omepral or Omez or Opal or Ozid or Rome 20 or Prilosec or Losec or Ulcozol or Segazole or Zegacid or Zegerid or Losepine).mp. [mp = title, abstract, subject headings, heading word, drug trade name, original title, device manufacturer, drug manufacturer, device trade name, keyword)
4. (73590-58-6 or 95510-70-6).mp.
5. or/1-4
6. esomeprazole.mp. or exp esomeprazole/ or exp esomeprazole strontium/
7. esome$.mp.
8. (217087-09-7 or 934714-36-0).mp.
9. (Nexium or Essocam or Esomezol or Racipher or Opton or Neptor or Nexemezol).mp. [mp = title, abstract, subject headings, heading word, drug trade name, original title, device manufacturer, drug manufacturer, device trade name, keyword)
10. or/6-9
11. cohort studies.mp. or exp cohort analysis/
12. exp case control study/ or case–control.mp.
13. (randomized controlled trial or random$).mp.
14. 11 or 12 or 13
15. 5 and 10
16. 14 and 15
